# P56 Azathioprine-induced alopecia totalis and knuckle hyperpigmentation in a child with uveitis: test before you leap!

**DOI:** 10.1093/rap/rkae117.087

**Published:** 2024-11-05

**Authors:** Prabal Barman, Sabia Handa, Ankur Kumar Jindal, Reema Bansal

**Affiliations:** Paediatric Allergy Immunology Unit, Department of Paediatrics, Advanced Paediatrics Centre, PGIMER, Chandigarh, India; Department of Ophthalmology, Advanced Eye Centre, PGIMER, Chandigarh, India; Paediatric Allergy Immunology Unit, Department of Paediatrics, Advanced Paediatrics Centre, PGIMER, Chandigarh, India; Department of Ophthalmology, Advanced Eye Centre, PGIMER, Chandigarh, India

## Abstract

**Introduction:**

Azathioprine is a common disease-modifying anti-rheumatic drug used in non-infectious uveitis. Although unusual, it has been associated with myelosuppression in genetically predisposed individuals who exhibit mutations in thiopurine S‐methyltransferase (*TPMT*) and nucleoside diphosphate‐linked moiety X‐type motif 15 (*NUDT15*) genes. Alopecia secondary to azathioprine treatment is rare, and is an early marker of azathioprine-induced myelosuppression. It usually precedes the onset of myelosuppression, but can also occur simultaneously with pancytopenia. Knuckle pad hyperpigmentation is strongly associated with megaloblastic anemia. We report a patient of panuveitis with both alopecia totalis and knuckle pad hyperpigmentation secondary to azathioprine-induced myelosuppression.

**Case description:**

A 13-year-old girl presented with acute-onset pain, redness and blurred of both eyes. Ophthalmological evaluation revealed bilateral granulomatous panuveitis. Visual acuity was 6/12 in both eyes, and anterior chamber had 2+ cells. Extensive evaluation for infective and autoimmune causes did not yield any aetiology. Following laboratory tests, a diagnosis of non-infectious panuveitis was made, and oral corticosteroids (2 mg/kg followed by taper) were started. As steroid sparing agents, subcutaneous methotrexate (15 mg/m^2^/week) and azathioprine (2 mg/kg) were also started after a normal baseline hemogram [Hb: 132 g/L, total leucocyte count (TLC) 12 x 10^9^/L and platelet count: 310 x 10^9^/L].

Two weeks later, she presented with complete loss of hair (alopecia totalis) and knuckle pad hyperpigmentation. Hemogram revealed pancytopenia (Hb: 86 g/L, TLC: 1.7 x 10^9^/L and platelet count: 25 x 10^9^/L). Considering drug-induced myelosuppression, azathioprine was stopped. Subcutaneous injections of Granulocyte colony-stimulating factor (G-CSF) (300 day) were given for two weeks. Mycophenolate mofetil (1200 mg/m^2^/day) was added. Quantitative polymerase-chain reaction genotyping assay showed a homozygous variant in *NUDT15* gene (c.415 C>T). Normalization of hemogram was seen after two weeks (Hb: 120 g/L, TLC: 10.44 x 10^9^/L and platelet count: 199 x 10^9^/L). Reversal of hair loss and knuckle hyperpigmentation was nearly complete at five months and six weeks of follow-up, respectively. Panuveitis remained resolved on methotrexate and mycophenolate, with 6/6 vision and no recurrence at 16-months of follow up.

**Discussion:**

In India, the prevalence of *TPMT* mutations (heterozygous 1.2-10%, and homozygous <1%), and *NUDT15* mutations (heterozygous 11.6%, and homozygous 1.4%) is low, and the tests are expensive. In routine clinical practice, monitoring of blood counts is recommended in patients on azathioprine (two weekly initially, and then four weekly).

Both TPMT and NUDT15 enzymes influence metabolism of azathioprine. Their polymorphisms result in accumulation of active 6-mercaptopurine, which gets converted to 6-thioguanine nucleotide metabolites, producing cytotoxic effects. As a result of disruption in mitotic activity of actively growing hair (anagen), more catagen follicles are seen than anagen, leading to hair loss. While massive hair loss is well recognized secondary to azathioprine, there is no report of knuckle pad hyperpigmentation as a direct adverse reaction of azathioprine. Both knuckle pad hyperpigmentation and azathioprine toxicity are associated with megaloblastic anemia. Knuckle pad hyperpigmentation may be explained by vitamin B_12_deficiency, with decreased intracellular glutathione. As a result, melanin deposition increases due to loss of tyrosinase inhibition. In addition, melanocyte pigmentary incontinence may also contribute to knuckle pad hyperpigmentation in such cases.

**Key learning points:**

• While alopecia is often massive, knuckle pad hyperpigmentation may often be overlooked as a clinical sign in patients with azathioprine-induced megaloblastic anemia. Our case highlights the significance of two simple clinical cutaneous signs (alopecia totalis and knuckle pad hyperpigmentation) as clinical indicators of impending, and potentially fatal complication of pancytopenia secondary to azathioprine. In developed countries, genetic analysis is often performed for identifying variants in *TPMT* and *NUDT15* genes before initiation of azathioprine. However, in resource-constrained settings, regular and close monitoring of hemogram, and prompt identification of clinical clues such as alopecia and knuckle pad hyperpigmentation may be a practical method to anticipate azathioprine-induced toxicity including myelosuppression.

